# Validation of the English and simplified Mandarin versions of the Fear of Progression Questionnaire – Short Form in Chinese cancer survivors

**DOI:** 10.1186/s40359-020-0374-0

**Published:** 2020-01-31

**Authors:** Rathi Mahendran, Jianlin Liu, Sangita Kuparasundram, Konstadina Griva

**Affiliations:** 1 Department of Psychological Medicine, National University of Singapore, NUHS Tower Block, Level 9, 1E Kent Ridge Road, Singapore, 119228 Singapore; 2 Academic Development Department, Duke-NUS Medical School, 8 College Road, Singapore, 169857 Singapore; 3 Institue of Mental Health, Buangkok Green Medical Park, 10 Buangkok View, Singapore, 539747 Singapore; 4 SingHealth Residency, Ministry of Health Holdings, 1 Maritime Square, Singapore, 099253 Singapore; 5 Lee Kong Chian School of Medicine, Nanyang Technological University, 59 Nanyang Drive, Experimental Medicine Building, Singapore, 636921 Singapore

**Keywords:** Fear of progression questionnaire, Validation, Simplified mandarin version

## Abstract

**Background:**

The fear of illness progression is common amongst those with chronic illnesses including cancers, and contributes to high psychological morbidity. Research in Asia on such fears however, is limited by a paucity of validated measurement instruments. Amongst the many available instruments, the Fear of Progression Questionnaire has a high quality rating, an important consideration in its selection. This study developed a simplified Mandarin version of the Fear of Progression Questionnaire – Short Form (FoP-Q-SF), and validated the English and Mandarin versions for use in Chinese populations.

**Methods:**

The translation to a simplified Mandarin version was through a forward-backward translation with emphasis on conceptual and cultural equivalence. Cancer survivors (*N* = 341) completed a self-report questionnaire, the Fear of Progression Questionnaire – Short Form, other measures of fear of progression, depression, anxiety, and quality of life. Reliability and criterion validity were assessed, and the factor-structure was replicated with a confirmatory factor analysis.

**Results:**

The Fear of Progression Questionnaire – Short Form demonstrated high internal and test-retest reliability. Criterion validity was also demonstrated through convergent, concurrent and discriminant validity. The factor structure was supported and replicated. The goodness-of-fit indices of the original model indicated some misfit, which could be adequately addressed by freeing five parameters in the error covariance matrix, without changing the one-factor structure.

**Conclusions:**

The Fear of Progression Questionnaire – Short Form is a reliable and valid measure of fear of progression applicable to a mixed-cancer survivor population in Singapore. The simplified Mandarin version of the questionnaire will be useful in other parts of Asia and for Chinese migrants in the West, further extending the use of this questionnaire.

## Background

One of the many concerns of patients with chronic diseases is that their illness will progress, which is a recognised realistic threat from their illness [1]. This fear has been examined in various disease groups, ranging from rheumatic diseases, parkinson disease, diabetes and chronic obstructive pulmonary disease, to cancers [2]. In cancer patients the focus of worry is that the illness will progress or metastasise, while amongst cancer survivors, it is that the illness will recur [3]. Although the term fear of recurrence was developed in relation to cancer patients’ fears, it is widely accepted that fear of progression and fear of recurrence share similar concepts with a recent expert panel integrating this as “Fear, worry, or concern about cancer returning or progressing” [4–6].

A recent systematic review of fear of recurrence and disease progression in long-term cancer survivors found that most survivors experience fear of recurrence at modest intensity with no significant change over time [7]. It is thus important that valid and effective instruments are available for assessment of these patient experiences. Of the various instruments available to assess fears of illness progression, the Fear of Progression Questionnaire (FoP –Q) has a high quality rating [8]. Although it was developed to measure fear of illness progression in chronically ill patients such as those with diabetes mellitus, rheumatic diseases and patients with systemic sclerosis, it has been mostly used with cancer patients [9, 10].

The 43-item FoP-Q comprises five subscales: affective reactions (13 items), partnership/family (7 items), occupation (7 items), loss of autonomy (7 items), and coping with anxiety (9 items) [9]. The Fear of Progression Questionnaire – Short Form (FoP-Q-SF), consisting of 12 items with four of the five subscales (excluding coping), was later developed and showed adequate reliability (α = 0.87) and validity in a sample of breast cancer patients and in a mixed-cancer survivor population, both in Germany [11, 12].

Apart from a Korean version of the FoP-Q developed and applied to cancer survivors and a Dutch translation used in a study of patients with systemic sclerosis, the scale has not been widely used [10, 13]. It has not been validated or translated for use in other Asian countries. Notably, there is no validated simplified Mandarin version of the FoP-Q-SF, which would be useful in China, parts of East and Southeast Asia, as well as amongst Mandarin-speaking migrant populations in the West, who use simplified Mandarin. In view of current gaps in the literature, the present study aimed to (1) develop a simplified Mandarin version of the FoP-Q-SF, and (2) validate the FoP-Q-SF for use in Chinese cancer populations. The validation of the FoP-Q-SF involved an examination of its reliability (i.e., internal consistency and test-retest reliability) and criterion validity (i.e., convergent validity, concurrent validity, divergent validity, and discriminant validity), as well as the confirmation of the factor structure in relation to the original model [9].

## Methods

### Procedures

#### Participants

As part of a large cross-sectional study examining the post-treatment fear of cancer recurrence (FCR) using the Fear of Cancer Recurrence Inventory (FCRI), potential participants were approached during their follow-up appointment at a cancer centre in Singapore (National University Cancer Institue Singapore, NCIS). The FoP-Q-SF was an additional instrument used in that study to validate the FCRI. Potential participants were recruited if they met the following inclusion criteria: (a) a cancer diagnosis; (b) at least one-year post-treatment (surgery, chemotherapy and/ or radiotherapy); (c) Singapore citizens or permanent residents between 21 to 84 years old; (d) ability to read and understand either English or Mandarin. The study received ethics approval from the National Healthcare Group Domain Specific Review Board (Reference: 2015/00003), and informed consent was obtained from all participants. 420 participants were recruited between February 2015 to June 2016, and 341 (81.2%) participants returned the questionnaire. Of these, 278 (81.5%) completed the FoP-Q-SF again two weeks later.

### Measures

#### Socio-demographic and medical variables

Participants completed a questionnaire on socio-demographic (gender, age, race, marital status, education, occupation), and medical variables (cancer type and stage, and cancer treatment received).

#### Fear of progression questionnaire – short form (FoP-Q-SF)

With permission from the developer of the FoP-Q, two independent staff did forward translations of the 12-item FoP-Q-SF, after which a reconciled version in simplified Mandarin was produced [9]. The approach taken in the translation was to emphasize conceptual rather than literal translations and to use natural and acceptable language that is simple and concise [14]. A bilingual group then reviewed this document and referenced a traditional Mandarin version that the developer had provided (Herschbach, personal communications), to produce a complete translated simplified version of the FoP-Q-SF.

An independent reviewer who was blinded to the original English version, then back translated the simplified Mandarin version. Emphasis was again on conceptual and cultural equivalence rather than linguistic equivalence. Comparisons and reviews were done to produce a satisfactory version in simplified Mandarin. Pre-testing was with five bilingual patients who reviewed both versions independently to assess the clarity, appropriateness of wording and acceptability of the translated questionnaire. All the iterations resulted in a final version of the FoP-Q-SF in simplified Mandarin.

Participants completed either the English or Mandarin versions of the FoP-Q-SF [11]. The items were rated on a Likert-type scale from one (never) to five (very often), which were summed to produce a total FOP score, with higher scores indicating a higher fear.

#### Other measures related to FOP

The FCRI examined the FCR in cancer patients [15]. Participants rated the 42 items on a Likert-type scale from zero (not at all or never) to four (a great deal or all the time) across seven components: the presence of potential stimuli activating FCR; the presence and severity of intrusive thoughts or images associated with FCR; the potential consequences of FCR; the level of self-criticism towards FCR intensity; and the coping strategies associated with FCR. One item was reverse-scored, and the sum of all items produced a total FCR score, with higher scores indicating higher levels of FCR. Similarly, the Fear of Recurrence Questionnaire (FRQ) measured the cancer patient’s fear of the probability of illness recurrence [4]. The items were rated on a Likert-type scale from one (strongly agree) to five (strongly disagree). Half the items were reverse-scored, and the sum of all 22 items yielded a total score, with higher scores indicating higher fear. The English and Mandarin versions of the FCRI has been validated and reported by the investigators [16].

#### Anxiety and depression

The 14-item Hospital Anxiety and Depression Scale (HADS) was measured through a four-point Likert-type scale, and the sum of all items produced a total distress score (HADS-Total) [17]. The items could also be loaded onto two separate subscales – anxiety (HADS-A) and depression (HADS-D). Higher scores indicated higher levels of distress.

#### Quality of life (QoL)

Participants completed the short-version of the World Health Organization Quality of Life Instrument short version (WHOQOL-BREF) [18]. The 26 items, through a four-point Likert-type scale, measured overall QoL, and four other domains of QoL – physical, psychological, social relations, and environment. Scores for each subscale were summed and transformed to range from zero to 100, and higher scores indicated a higher self-perceived QoL.

### Statistical Analyses

#### Reliability

Cronbach alpha coefficients and corrected item-total correlations were calculated to assess the internal consistency of the FoP-Q-SF. A Cronbach alpha coefficient of more than .70 supported satisfactory internal consistency [19]. Test-retest reliability was assessed through calculating Pearson correlations between the FoP-Q-SF scores at both time-points.

#### Criterion validity

To support convergent validity, it was hypothesized that the FoP-Q-SF would be strongly correlated with other instruments measuring the fear of progression in cancer survivors (FCRI and FRQ). Concurrent validity was examined through comparing FoP-Q-SF with measures of emotional distress. As a related but distinct construct from emotional distress, it was expected that FoP-Q-SF would be moderately correlated to measures examining emotional distress, especially anxiety [9]. Divergent validity was examined through correlations between FOP and its potential consequences – quality of life (QoL). Negative correlations were expected between FoP-Q-SF and measurements of QoL in view of previous empirical findings; persistent fear of illness progression is associated with pathological worry, which may have a detrimental impact on QoL [7, 20]. Discriminant validity was examined through associations between FoP-Q-SF and basic socio-demographic and medical variables. Specifically, a significant weak, negative correlation was expected between FoP-Q-SF and survivor’s age [10, 11]. Tests of Pearson correlations or one-way analyses of variance (ANOVAs) were conducted, with Bonferroni corrections employed for each set of comparisons to reduce family-wise Type I errors. The strength of the correlational relationship was determined based on Cohen’s criteria: *r* = 0.1 (weak), *r* = 0.3 (moderate), and *r* = 0.5 (strong) [21]. Additional significant tests based on Meng and colleagues’ recommendations were adopted to compare the level of strength in the association between the scores of the questionnaires [22].

#### Model structure confirmation

Confirmatory factor analyses (CFA) were conducted to confirm the factor structure of the FoP-Q-SF. Model fit was examined using three indices: (a) Comparative Fit Index (CFI) ≥ .90, (b) Root-Mean-Square-Error of Approximation (RMSEA) ≤ .06, and (c) Standardised Root Mean Square Residual (SRMR) ≤ .08 [23]. Based on modification indices, error variances were allowed to covary in order to improve model fit.

The CFA was conducted using Mplus version 6.12 [24]. All other statistical analyses were conducted with SPSS 23.

## Results

### Socio-demographic and medical variables

Table 1 presents the descriptive statistics. The mean age of the participants was 55.32 (±11.13). A majority of the participants were female (80.4%). Breast cancer (37.0%) and gynaecological cancers (27.3%) were the most prevalent cancer types. Most participants had early stage cancer (57.4%). In terms of treatment, 58.1% had undergone chemotherapy, 44.9% had undergone radiotherapy, and 71.6% of the participants had undergone surgery.

**Table 1:** Descriptive Data

Socio-demographic and Medical Variables	Mean (SD)/ N (%^a^)
Age	55.32 (11.13)
Gender	
Male	67 (19.6)
Female	274 (80.4)
Race	
Chinese	271 (79.5)
Malay	37 (10.9)
Indian	20 (5.9)
Others	12 (3.5)
Marital Status	
Single	49 (14.4)
Married	243 (71.3)
Divorced/ Separated	23 (6.7)
Widowed	23 (6.7)
Education	
No Formal Education	11 (3.2)
Primary Education	59 (17.3)
Secondary/ GCE ‘N’/‘O’ Levels/ Vocational Education	152 (44.6)
GCE ‘A’ Levels/ Polytechnic Diploma	58 (17.0)
Bachelor’s Degree	47 (13.8)
Postgraduate Education	10 (2.9)
Occupation	
Full-time	144 (42.2)
Part-time	49 (14.4)
Retired	56 (16.4)
Homemaker	82 (24.0)
Cancer Type	
Breast	126 (37.0)
Gynecological	93 (27.3)
Gastro-intestinal	47 (13.8)
NPC/ Throat/ Oral	14 (4.1)
Hemotological/ Leukemia/ Lymphoma/ Myeloma	11 (3.2)
Lung	11 (3.2)
Brain	2 (0.6)
Pancreas	1 (0.3)
Others	12 (3.5)
Multi-site	14 (4.1)
Cancer Stage	
Early (Stages 0–2)	196 (57.4)
Late (Stages 3–4)	90 (26.4)
Underwent Chemotherapy	
Yes	198 (58.1)
No	135 (39.6)
Underwent Radiotherapy	
Yes	153 (44.9)
No	180 (52.8)
Underwent Surgery	
Yes	244 (71.6)
No	89 (26.1)

^a^ Percentages might not add up to 100% due to missing data or rounding difference

### Reliability

Both versions of the FoP-Q-SF demonstrated high internal reliability (English: α = .87–.88; Mandarin: α = .88 at both time-points), and test-retest reliability (English: *r* = .85, *p* < .01; Mandarin: *r* = .83, *p* < .01). The two versions were thus combined for further analyses. Overall, internal reliability (α = .88–89), and test-retest reliability was supported (*r* = .85, *p* < .01).

### Criterion validity

The criterion validity of the FoP-Q-SF is presented in Table 2. The FoP-Q-SF was strongly correlated with other measures of FOP in cancer patients – the FCRI (*r* = .66, *p* < .001) and the FRQ (*r* = .64, *p* < .001). The significance tests revealed no significant differences between the correlation coefficients (*r* = .66 and *r* = .64) (95% Confidence Interval [CI]: − 0.07, 0.14).

**Table 2:** Criterion validity of the Fear of Progression Questionnaire – Short Form

Measures^a^	Cronbach’s Alpha	M (SD)	Correlations with FoP-Q-SF
FoP-Q-SF	.88	27.85 (9.17)	
FCRI	.95	61.3 (31.53)	.66**
FRQ	.90	69.74 (11.58)	.64**
HADS			
HADS-Total	.88	8.52 (6.54)	.55**
HADS-A	.77	5.02 (3.99)	.61**
HADS-D	.86	3.50 (3.32)	.35**
WHOQOL-BREF			
Overall Quality of Life	.67	60.00 (15.69)	−.28**
Physical Health	.79	68.71 (14.85)	−.32**
Psychological	.82	66.98 (15.07)	−.37**
Social Relationships	.72	64.94 (17.26)	−.26**
Environment	.87	66.22 (15.51)	−.20**

^a^ *FoP-Q-SF* Fear of Progression Questionnaire Short-Form, *FCRI* Fear of Cancer Recurrence Inventory, *FRQ* Fear of Recurrence Questionnaire, *HADS* Hospital Anxiety and Depression Scale, *WHOQOL-BREF* World Health Organization Quality of Life Instrument (Short version)

** *p* < .001

Strong correlations were demonstrated between the FoP-Q-SF and HADS-Total (*r* = .55, *p* < .001), and HADS-A (*r* = .61, *p* < .001), while moderate correlations were demonstrated with HADS-D (*r* = .35, *p* < .001). The significance tests revealed significant differences between the HADS-A (*r* = .61) and HADS-Total (*r* = .55) (95% CI: 0.04, 0.14), as well as between the HADS-A (*r* = .61) and HADS-D (*r* = .35) (95% CI, 0.24, 0.45).

Weak to moderate negative associations were found between FoP-Q-SF and various domains of the WHOQOL-BREF – overall QoL (*r* = −.28, *p* < .001), physical health (*r* = −.32, *p* < .001), psychological (*r* = −.37, *p* < .001), social relationships (*r* = −.26, *p* < .001), and environmental (*r* = −.20, *p* < .001). Weak to moderate negative associations were found between FoP-Q-SF and age (*r* = −.27, *p* < .001). The significance tests revealed no significant differences between the weak to moderate negative associations for QoL (*r* = −.28) and age (*r* = −.27) (95% CI: − 0.16, 0.14).

FoP-Q-SF scores differed based on ethnicity (*F*[3336] = 5.33, *p* < .01); Malay patients were more likely to have a higher score than Chinese patients (*p* < .01). FoP-Q-SF also differed based on occupation (*F* [3327] = 6.48, *p* < .001); patients working full-time were more likely to have a higher score than those working part-time (*p* < .05) or those who were retired (*p* < .001). Patients who underwent radiotherapy were more likely to have a higher FoP-Q-SF score than those who did not (*F* [1331] = 6.61, *p* < .05). FoP-Q-SF did not differ significantly based on gender, marital status, cancer type, cancer stage, or whether the patient underwent chemotherapy (*p*s ≥ .13).

### Confirmatory factor analyses

The initial model (χ^2^ [54, 273] = 211.100, *p* < .001) demonstrated an SRMR (.057) that met the criteria for an adequate model fit. However, the CFI (.87) was lower, and RMSEA (.103) was higher, than the recommended criteria, which indicated some misfit in the model. As such, the sources of misfit were examined. All parameter estimates and their corresponding standard errors were statistically significant (*p*s < .001). However, the modification indices suggested that the model fit could be improved if certain parameters in the error covariance matrix were freed, while leaving the original one-factor structure intact. As such, an additional 5 covariance parameters between the following pairs of items were added to the initial model: (1) Items 4 and 12 (Est. = .366, S.E. = .074, *p* < .001); (2) Items 1 and 8 (Est. = .177, S.E. = .036, *p* < .001); (3) Items 6 and 11 (Est. = .397, S.E. = .075, *p* < .001, 4) Items 6 and 7 (Est. = .198, S.E. = .059, *p* < .01); and (5) Items 1 and 2 (Est. = .127, S.E. = .039, *p* < .01). The revised model (χ^2^ [49, 273] = 87.47, *p* < .001) showed a CFI (0.97), RMSEA (.054) and SRMR (.039) that met the criteria for an adequate model fit. The post-hoc model fitting was therefore ceased. This model demonstrated an improvement in model fit as compared to the initial model). The goodness-of-fit for the initial and revised models are summarized in Table 3.

**Table 3:** Fit indices for the Fear of Progression Questionnaire – Short Form

Models tested and the items that error of covariance were released		Fit Indices ^a^				
		χ^2^	df	CFI	RMSEA	SRMR
Initial FoP-Q-SF		230.561	54	0.869	0.106	0.058
Item 4	Being afraid of becoming less productive at work	183.698	53	0.903	0.092	0.053
Item 12	Being afraid of not being able to work anymore					
Item 6	Being afraid of the possibility that the children could contract cancer	151.805	52	0.926	0.081	0.047
Item 11	Worrying about what will become of the family					
Item 1	Being afraid of disease progression	124.886	51	0.945	0.071	0.044
Item 8	Being afraid of no longer be able to pursue hobbies					
Item 1	Being afraid of disease progression	111.377	50	0.954	0.065	0.04
Item 2	Being nervous prior to doctor’s appointment or periodic examination					
Item 6	Being afraid of the possibility that the children could contract cancer	99.627	49	0.962	0.06	0.038
Item 7	Being afraid of relying on strangers for activities of daily living					

^a^ *CFI* Comparative fit index, *RMSEA* Root-mean-square error of approximation, *SRMR* standard root-mean-squared residual.

Criteria used: CFI ≥ .95; RMSEA ≤ .06; SRMR ≤ .08

## Discussion

The present study developed a simplified Mandarin version of the FoP-Q-SF. The reliability and validity of both the English and Mandarin versions of the FoP-Q-SF were established in a group of mixed-cancer survivors in Singapore, providing a validated tool that may be used to assess the FOP in cancer survivors.

The FoP-Q-SF demonstrated high internal reliability, with coefficients greater than the cut-off criteria of .70. High strong correlations were also demonstrated between T1 and T2 scores, supporting test-retest reliability. The results largely supported the criterion validity of the FoP-Q-SF. In line with expectations, the FoP-Q-SF was strongly correlated with other measures of FCR in cancer survivors and there were no differences in the strength of these associations. Concurrent validity was supported with significant strong correlations with psychological distress, especially anxiety; this is also supported by the stronger positive association with anxiety than with depression or HADS total scores. Divergent validity was supported with weak to moderate negative correlations with QoL. Discriminant validity was also demonstrated with weak to moderate negative associations with age. These weak to moderate negative associations did not differ in terms of magnitude, which further supports both divergent validity and discriminant validity respectively.

Overall, the factor structure of the FoP-Q-SF was supported and replicated in the present study. The goodness-of-fit indices of the original model indicated some misfit, which could be adequately addressed by freeing five parameters in the error covariance matrix, without changing the one-factor structure. It is conceptually and statistically acceptable to allow residuals to correlate as correlated errors are likely due to potential redundancy of item content [6, 16]. In the present study, item 4 (“being afraid of becoming less productive at work) and item 12 (“being afraid of not being able to work anymore”) may be perceived as similar concerns about occupational disruptions. On the other hand, item 6 (“being afraid of the possibility that the children could contract cancer”), item 7 (“being afraid of relying on strangers for activities of daily living”), and item 11 (“worrying about what will become of the family”) may be perceived as similar concerns about interpersonal relationships. In contrast to specific concerns about occupational disruptions and interpersonal relationships, item 1 (“being afraid of disease progression), item 2 (“being nervous prior to doctor’s appointment or periodic examination), and item 8 (“being afraid of no longer be able to pursue hobbies) may be perceived as general worries associated with disease progression. As noted by previous validation studies on other FCR measures, these minor adjustments to improve model fit do not have any implications on the administration of the scale [6].

### Limitations

The sample might not be representative of the mixed-cancer survivors population in multilingual Singapore, as participation was limited to those who were able to read either English or Mandarin; participants who were illiterate or those who were proficient in other languages were unable to participate. Second, the generalizability of the present study might be limited, as a majority of the participants were female, and had breast or gynaecological cancer. Third, patients who declined participation or did not return their questionnaires could potentially have significantly different levels of FOP, limiting the representativeness of the sample. As socio-demographic information were not collected from these participants, this prevented further statistical comparisons to determine if non-participants deferred significantly from participants.

## Conclusions

With medical advances in cancer detection and treatment, cancer survivorship has increased, and is expected to increase further [25]. High levels of fears of illness progression are associated with poorer quality of life and psychosocial well-being, highlighting a need to investigate dysfunctional levels of this fear amongst cancer survivors [7, 20]. The simplified Mandarin version of the FoP-Q-SF that has been produced will allow wider utilisation of this instrument in Mandarin-speaking populations. The study also validated the FoP-Q-SF in a mixed-cancer population in Singapore. As a short 12-item scale, this provides a potentially useful screening tool to assess levels of fear of illness progression in cancer survivors, and could enhance care for survivors.

There is potential to validate the English and simplified Mandarin version of the FoP-Q-SF for use in other chronic illness in Singapore and other Asian countries. This would extend a better understanding of fear of illness progression across different illnesses, and facilitate future research such as severity cut-offs and predictors of higher fear of illness progression. A recent study which found that the FoP-Q could be adapted for parental caregivers, further extends its usefulness [26].

## Data Availability

The data is available from the corresponding author on reasonable request and subject to Ethics Board approval.

## Abbreviations

ANOVA): Analyses of Variance
CFA: Confirmatory Factor Analysis
CFI: Comparative Fit Index
FCR: fear of cancer recurrence
FCRI: Fear of Cancer Recurrence Inventory
FoP-Q: Fear of Progression Qustionnaire
FoP-Q-SF: Fear of Progression Questionnaire – Short Form
FRQ: Fear of Recurrence Questionnaire
HADS – A: Hospital Anxiety and Depression Scale – anxiety
HADS – D: Hospital Anxiety and Depression Scale – depression
HADS: Hospital Anxiety and Depression Scale
NCIS: National University Cancer Institute Singapore
QoL: quality of life
RMSEA: Root-Mean-Square-Error of Approximation
SPSS: Statistical Package for Social Sciences
SRMR: Standardised Root Mean Squared Residual
WHOQOL-BREF: World Health Organization Quality of Life Instrument Short Version
